# A Comprehensive Biophysical Model of Ion and Water Transport in Plant Roots. III. Quantifying the Energy Costs of Ion Transport in Salt-Stressed Roots of *Arabidopsis*

**DOI:** 10.3389/fpls.2020.00865

**Published:** 2020-07-03

**Authors:** Kylie J. Foster, Stanley J. Miklavcic

**Affiliations:** Phenomics and Bioinformatics Research Centre, University of South Australia, Mawson Lakes, WA, Australia

**Keywords:** biophysical modeling, Cl^−^ transport, energy cost, energy efficiency, Na^+^ transport, salinity, salt stress

## Abstract

Salt stress defense mechanisms in plant roots, such as active Na^+^ efflux and storage, require energy in the form of ATP. Understanding the energy required for these transport mechanisms is an important step toward achieving an understanding of salt tolerance. However, accurate measurements of the fluxes required to estimate these energy costs are difficult to achieve by experimental means. As a result, the magnitude of the energy costs of ion transport in salt-stressed roots relative to the available energy is unclear, as are the relative contributions of different defense mechanisms to the total cost. We used mathematical modeling to address three key questions about the energy costs of ion transport in salt-stressed *Arabidopsis* roots: are the energy requirements calculated on the basis of flux data feasible; which transport steps are the main contributors to the total energy costs; and which transport processes could be altered to minimize the total energy costs? Using our biophysical model of ion and water transport we calculated the energy expended in the trans-plasma membrane and trans-tonoplast transport of Na^+^, K^+^, Cl^−^, and H^+^ in different regions of a salt-stressed model *Arabidopsis* root. Our calculated energy costs exceeded experimental estimates of the energy supplied by root respiration for high external NaCl concentrations. We found that Na^+^ exclusion from, and Cl^−^ uptake into, the outer root were the major contributors to the total energy expended. Reducing the leakage of Na^+^ and the active uptake of Cl^−^ across outer root plasma membranes would lower energy costs while enhancing exclusion of these ions. The high energy cost of ion transport in roots demonstrates that the energetic consequences of altering ion transport processes should be considered when attempting to improve salt tolerance.

## 1. Introduction

Soil salinity is a global problem affecting 1 billion ha of land, including 20% of all irrigated land (FAO and ITPS, 2015). Salinity reduces growth and yields in many agriculturally important plant species. The cause of this reduced growth and yield is an area of active research (Tyerman et al., 2019); however, it is already recognized that a reduction in energy production (e.g., resulting from reduced leaf area) and an increase in energy required for defense mechanisms may play critical roles (Munns and Gilliham, 2015; Munns et al., 2020a). For example, preventing cytosolic Na^+^ from accumulating to toxic levels is an important defense mechanism in roots that requires energy. While Na^+^ transport into the root symplast is a passive process driven by a favorable electrochemical potential difference (Munns and Tester, 2008; Maathuis et al., 2014), active efflux of Na^+^ out of the cytosol is required to prevent Na^+^ toxicity (Munns and Tester, 2008; Foster and Miklavcic, 2019). Na^+^ efflux to the apoplast is thought to occur via plasma membrane Na^+^/H^+^ antiporters in a secondary active process, which is powered by plasma membrane H^+^-ATPases (Munns and Tester, 2008; Foster and Miklavcic, 2019). Similarly, Na^+^ storage in vacuoles relies on secondary active Na^+^ transport via Na^+^/H^+^ antiporters, powered by tonoplast H^+^-ATPases and PPases (Maathuis et al., 2014). Transmembrane transport of other ions, such as Cl^−^ and K^+^, also requires ATP (Szczerba et al., 2009; Li et al., 2017a). If less ATP was required for these ion transport processes, more energy would be available for plant growth and grain production (Munns and Gilliham, 2015). Hence, when breeding crops with improved salt tolerance it would be beneficial to understand the energy demands resulting from salt stress, and to identify ways by which plants may reduce these energy requirements.

The amount of energy required for ion transport in salt-stressed roots in currently unclear. Energy cost estimates by Malagoli et al. (2008), Britto and Kronzucker (2009), and Kronzucker and Britto (2011) suggest that the amount of energy required for Na^+^ efflux out of outer root cells exceeds reasonable estimates of the amount of ATP available from root respiration for a range of plant species and external NaCl concentrations. In addition, the energy cost of Cl^−^ influx into the outer root (Teakle and Tyerman, 2010) and energy cost of transporting H^+^ across tonoplasts (Shabala et al., 2019) have each been estimated to be of a similar order of magnitude to the entire amount of ATP available. Given that roots also require ATP for maintenance, growth and other ion transport processes these estimates are unfeasible. In contrast, Munns et al. (2020b) estimated that Na^+^ efflux from wheat and barley roots growing in 150 mM NaCl would require 3–24% of the total energy available from root respiration. Similarly, Munns et al. (2020a) estimated that the transport of both Na^+^ and Cl^−^ from the external medium to the root xylem would require approximately 11–46% of the energy available from root respiration for a wheat plant grown in 150 mM NaCl. Each of these energy cost calculations are based on assumptions about ion fluxes that are not well-known or understood, with different assumptions leading to vastly different energy cost estimates. These ion fluxes are based on radioactive tracer flux measurements and a possible explanation for the unrealistically high energy estimates is that the radioactive tracer flux data do not accurately reflect transmembrane fluxes (Malagoli et al., 2008; Munns et al., 2020b). Alternatively, the mismatch between energy supply and demand could suggest that current understanding of Na^+^, Cl^−^, and H^+^ transport in roots may need to be revised. In addition, the amount of ATP available is uncertain and is affected by many factors (Munns et al., 2020a). Although the disparity between the energy costs of Na^+^ transport and the available energy was identified over a decade ago (Malagoli et al., 2008), it is still unclear if this conundrum is due to a problem with trans-plasma membrane flux measurements or an incomplete understanding of ion transport.

Using existing experimental methods it is impossible to measure ion fluxes at the level of detail required to accurately quantify and compare the energy costs of different salt stress response mechanisms. For instance, it is currently unknown which ion transport steps have the highest energy costs. Such a comparison could advise us on which transport processes to target to improve salt tolerance (Munns and Gilliham, 2015; Munns et al., 2020a). The required level of detail can be achieved using mathematical modeling (Arsova et al., 2020; Munns et al., 2020a). For example, we have previously used modeling to demonstrate that anatomical changes to roots can affect energy costs (Arsova et al., 2020; Munns et al., 2020a), and that day/night changes in transpiration can also affect energy costs in salt-stressed roots (Arsova et al., 2020). However, these modeling efforts did not identify the transport processes that were the key contributors to overall energy costs.

To improve our understanding of the energy requirements involved in the salt stress response we applied our model of ion and water transport in a salt-stressed root (Foster and Miklavcic, 2017, 2019) to address three questions: Are the energy costs of ion transport in salt-stressed roots feasible given current estimates of available ATP and our current understanding of the key transport mechanisms involved? Which transport processes are the most significant contributors to the total energy cost of ion transport? What changes to ion transporter activities and channel permeabilities lead to favorable salt stress responses while also minimizing total energy costs? To address these questions we calculated the amount of ATP required to transport Na^+^, K^+^, H^+^, and Cl^−^ across plasma membranes and tonoplasts. Our approach allowed us to estimate the energy costs of many transport steps, including interactions between ions. Our model parameters were based on *Arabidopsis thaliana* roots and were obtained by comparisons with experiments (Foster and Miklavcic, 2019). These parameters are independent of radioactive tracer flux measurements across the root surface.

We found that our calculated energy expenditure exceeded the amount of energy estimated to be available from root respiration for high external NaCl conditions. Na^+^ efflux and Cl^−^ influx across the plasma membranes of the outer root cells were the main contributors to the overall total energy cost. We found, however, that these costs could be reduced, while still excluding Na^+^ from the root symplast, by limiting the passive uptake of Na^+^ and the active uptake of Cl^−^.

## 2. Materials and Methods

### 2.1. Model Description

**Figure 1:**
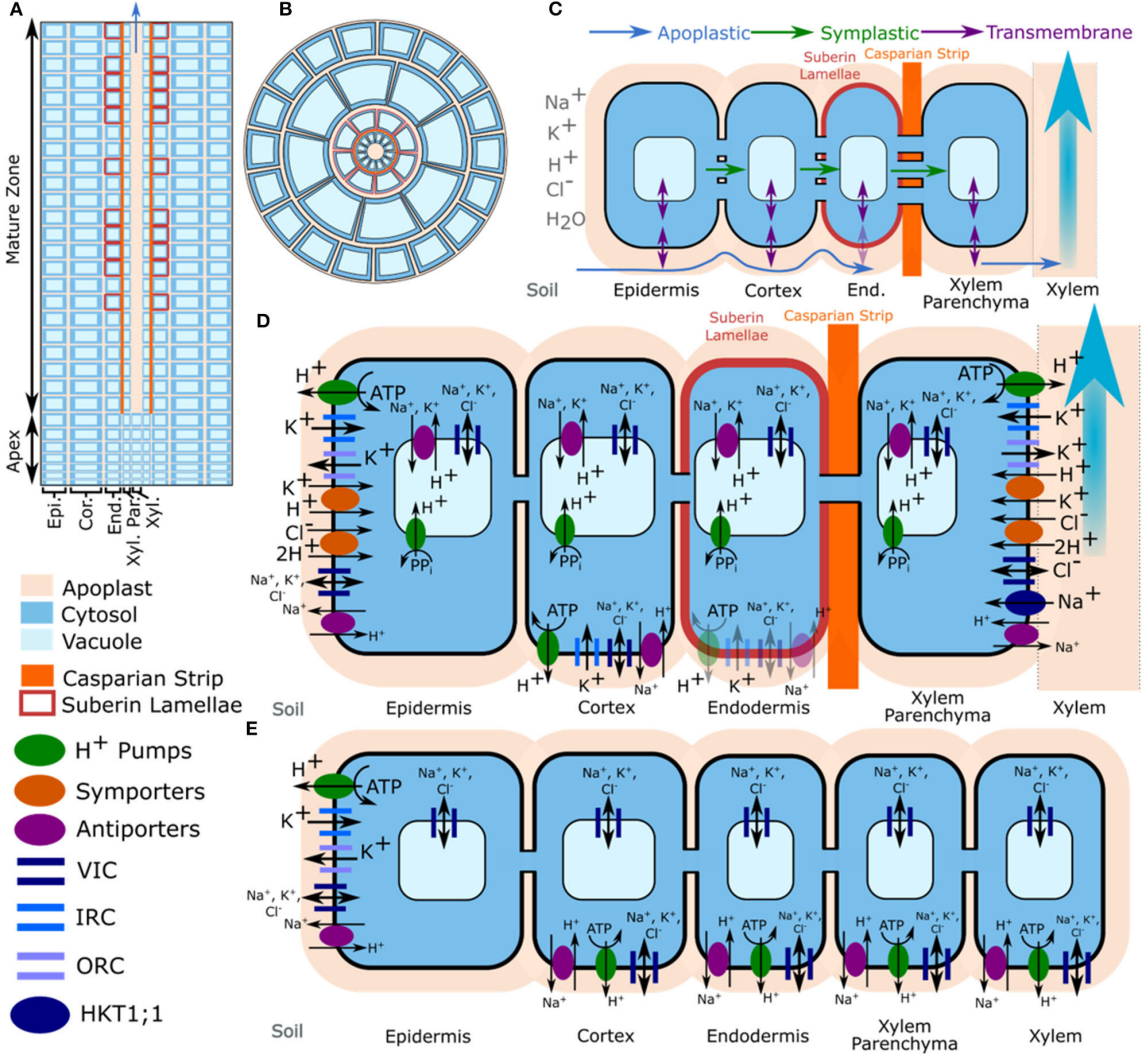
Model root structure, transport pathways, and tissue specific location of transport proteins. **(A)** Axial cross-section of the model root cylinder showing the discretisation of the root, and the division into two developmental zones. **(B)** Top view of the model root showing the radial discretisation into different root tissue regions and partitioning of apoplastic, cytosolic, and vacuolar compartments. Although they are not shown in this view, the model includes plasmodesmata. **(C)** Simulated ion and water transport pathways. The simulations also included axial symplastic and apoplastic fluxes (not shown). **(D)** Spatial distribution of transporters and channels in the mature root. **(E)** Spatial distribution of transporters and channels in the apex. Multiple small vacuoles that may be present in the meristem are simulated as a single larger vacuole. Trans-plasma membrane transport in the mature endodermis was assumed to occur only in cells lacking suberin lamellae (e.g., passage cells). The distributions of K^+^ voltage dependent channels and K^+^/H^+^ symporters were obtained from the literature (Lagarde et al., 1996; Gaymard et al., 1998; Ivashikina et al., 2001; Desbrosses et al., 2003; Gierth et al., 2005). The location of plasma membrane Na^+^/H^+^ antiporters was based on findings in Foster and Miklavcic (2019), while the location of tonoplast Na^+^/H^+^ antiporters was based on findings in Shi et al. (2002). Adapted from Foster and Miklavcic (2019).

The model is described in detail in Foster and Miklavcic (2017) and Foster and Miklavcic (2019), and is summarized in Figure 1. The complete set of model equations are provided in the Supplementary Material of Foster and Miklavcic (2019)^1^. In brief, the anatomical model root is assumed to be a flat-ended cylinder, with geometry and root tissue arrangement based on *Arabidopsis* roots (Foster and Miklavcic, 2013, 2014, 2016). The root is divided in the radial direction into five tissue regions representing the: epidermis, cortex, endodermis, xylem parenchyma and xylem. In the axial direction the root is divided into 30 layers each corresponding to approximately the height of a cell. The root is also split axially into two developmental zones: an apex, which does not contain functional xylem; and a mature zone, which contains functional xylem as well as a Casparian strip and suberin lamellae in the endodermis. The model elements formed by discretising the root in the radial and axial directions are further subdivided into apoplastic, cytosolic and vacuolar compartments (see Figure 1). The cytosols of adjacent cells are connected by plasmodesmata. The apoplastic and cytosolic compartments are separated by plasma membranes, while the cytosolic and vacuolar compartments are separated by tonoplast membranes.

The model includes axial and radial, apoplastic and symplastic transport, as well as transmembrane transport of ions (Na^+^, K^+^, Cl^−^, and H^+^) and water (Foster and Miklavcic, 2015). Binding of mobile cations to the bound anions present in the apoplast is also included. Apoplastic and symplastic ion transport is driven by electrochemical diffusion and convection (modeled using an extended Nernst-Planck equation). Ion transport across both tonoplasts and plasma membranes is assumed to occur via: Na^+^, K^+^, and Cl^−^ permeable channels; Na^+^/H^+^ antiporters; and H^+^ pumps. In addition, K^+^ is transported across tonoplasts via K^+^/H^+^ antiporters and across plasma membranes via inward rectifying K^+^ channels (IRC), outward rectifying K^+^ channels (ORC), and K^+^/H^+^ symporters. The model includes apoplastic K^+^ concentration dependent gating of ORCs (Ivashikina et al., 2001; Johansson et al., 2006). Cl^−^/2H^+^ symporters are also present on the plasma membranes and HKT1;1 transports Na^+^ across xylem parenchyma plasma membranes. The effect of external Ca^2+^ on the permeability of non-selective cation channels (NSCCs) (Demidchik and Tester, 2002) is also simulated. The transporters and channels are assumed to be non-uniformly distributed across the root tissue regions as shown in Figure 1. Due to the difficulties in distinguishing between the contributions of the two types of tonoplast H^+^ pumps (V-ATPase and PPase) we assume there is only a single type of H^+^ pump operating on the tonoplast, which represents a “composite” of the fluxes through the tonoplast V-ATPase and PPase.

Water transport was modeled using non-equilibrium thermodynamics. Water transport across plasma membranes and through the symplast is driven by osmotic and hydraulic pressure differences. Apoplastic water transport is driven by hydraulic pressure differences alone, while water flow across tonoplasts is driven by osmotic pressure differences only.

The system of differential and algebraic equations resulting from the physical model described above were solved numerically in MATLAB (see Foster and Miklavcic, 2017 for details). Unless otherwise stated all simulations were conducted with a pressure boundary condition of −0.3 MPa at the top of the root, with the external medium containing 100 mM NaCl, 1 mM KCl, 0.1 mM Ca^2+^, and having a pH of 5. The model parameters are provided in Foster and Miklavcic (2019). These parameters were obtained by fitting the model to experimental data measured for *Arabidopsis* wild-type and *sos1* roots (including measurements of Na^+^ and K^+^ root contents; Na^+^, K^+^ and anion xylem concentrations; Na^+^ fluxes to the shoot; and epidermal membrane potentials) or were obtained from the literature (Foster and Miklavcic, 2019).

### 2.2. Energy Supply and Demand Calculations

Our model simulations provide time-dependent Na^+^, K^+^, H^+^, and Cl^−^ fluxes across each individual root cell plasma membrane and tonoplast. The equations for these fluxes are provided in the Supplementary Material of Foster and Miklavcic (2019)^1^. To convert these ion fluxes to the amount of ATP consumed we adopted the following transporter stoichiometries: 1 Na^+^: 1 H^+^ for the plasma membrane and tonoplast antiporters (Darley et al., 2000; Qiu et al., 2003); 1 K^+^: 1 H^+^ for the tonoplast antiporters; 1 K^+^: 1 H^+^ and 1 Cl^−^: 2 H^+^ for the plasma membrane symporters (Beilby and Walker, 1981; Felle, 1994; Maathuis et al., 1997); 1 H^+^ transported per ATP hydrolyzed for the plasma membrane H^+^ pumps (Briskin and Reynolds-Niesman, 1991; Briskin et al., 1995); and 2 H^+^ transported per ATP hydrolyzed for the tonoplast H^+^ pump (Davies et al., 1994). As mentioned above, H^+^ fluxes through the model tonoplast pump represent a composite of the fluxes through the V-ATPase and PPase, and therefore cannot be used to calculate the individual energy costs for each type of pump. Instead, an upper bound for the ATP cost of ion transport across the tonoplast membranes was calculated by assuming that all model H^+^ tonoplast fluxes were transported via V-ATPase.

It is important to recognize that the energy calculations based on a stoichiometry of one H^+^ translocated for every one molecule of ATP consumed by the plasma membrane pumps is a conservative ratio supported by some previous experimental works (Venema and Palmgren, 1995; Kurimoto et al., 2004; Malagoli et al., 2008; Munns et al., 2020a). As this ratio is assumed here to apply uniformly across the root, our results could be viewed as providing more of a worst-case scenario. This viewpoint may be supported by the existence of evidence of a more favorable stoichiometric ratio, such as 3:1, occurring in some situations (Kerkeb et al., 2002). This would result in a positive shift in the ensuing energy balance. On the whole, though, it is reasonable to acknowledge that the H^+^:ATP stoichiometry is more likely plant, organ and tissue specific, as well as being dependent on the level of stress to which a plant is subjected.

The total cost of transport via H^+^ pumps across all root cell membranes (in μmol ATP g^−1^ root FW h^−1^) was determined using the total unidirectional (influx/efflux) fluxes of H^+^ via both plasma membrane and tonoplast H^+^ pumps, combined with the H^+^ to ATP stoichiometries identified above:

(1) $$\begin{array}{l} {\text{total cost of H}}^{+}\text{ transport}\\ =\frac{1}{\text{root mass}}\overset{\text{all cells}}{\sum }\left({\text{H}}^{+}\text{fluxes via plasma membrane pumps}\right)\\ \;+\frac{1}{2\times \text{root mass}}\overset{\text{all cells}}{\sum }\left({\text{H}}^{+}\text{fluxes via tonoplast pumps}\right). \end{array}$$

Our model framework explicitly ensures that electroneutrality and mass balance are maintained. Model fluxes via each type of transporter are based on the relevant electrochemical potential gradients, with electroneutrality used to determine the membrane potentials (see Foster and Miklavcic, 2019). Hence, the interconnected nature of membrane potentials and ion fluxes is included in the model, with the ion flux equations including membrane potentials and the membrane potential equations including ion fluxes. As a result, although Equation (1) does not explicitly include the contribution of the H^+^ pumps to the transmembrane electric potentials, this effect is implicitly included in the framework of our model.

At steady state, the efflux of H^+^ out of the cytosol across tonoplasts via antiporters must equal the influx of H^+^ via tonoplast pumps. Similarly, the influx of H^+^ across plasma membranes via symporters and antiporters must equal the efflux of H^+^ via plasma membrane pumps. Therefore, at steady state, total ATP cost of transmembrane ion transport in the model is given by Equation (1). This cost was split into the cost of transporting individual ions (Na^+^, K^+^, Cl^−^) by calculating the fluxes of these ions through each transporter and taking into account the appropriate stoichiometries:

(2) $$\begin{array}{l} {\text{cost of Na}}^{+}\text{ transport}\\ =\frac{1}{\text{root mass}}\overset{\text{all cells}}{\sum }\left({\text{Na}}^{+}\text{ fluxes via plasma membrane antiporters}\right)\\ \;+\frac{1}{2\times \text{root mass}}\overset{\text{all cells}}{\sum }\left({\text{Na}}^{+}\text{ fluxes via tonoplast antiporters}\right); \end{array}$$

(3) $$\begin{array}{l} {\text{cost of Cl}}^{-}\text{ transport}\\ =\frac{2}{\text{root mass}}\overset{\text{all cells}}{\sum }\left({\text{Cl}}^{-}\text{ fluxes via plasma membrane symporters}\right); \end{array}$$

(4) $$\begin{array}{l} {\text{cost of K}}^{+}\text{ transport}\\ =\frac{1}{\text{root mass}}\times \overset{\text{all cells}}{\sum }\left({\text{K}}^{+}\text{ flux via plasma membrane symporters}\right)\\ \;+\frac{1}{2\times \text{root mass}}\times \overset{\text{all cells}}{\sum }\left({\text{K}}^{+}\text{ flux via tonoplast antiporters}\right). \end{array}$$

In addition, to identify which transport mechanisms were particularly costly, and in which regions, we calculated the energy costs of several individual transport processes by combining fluxes and transport stoichiometries, including:

(5) $$\begin{array}{l} \text{cost of }{\text{Na}}^{+}\text{ transport across outer root cell plasma membranes}=\\ \;\frac{1}{\text{root mass}}\times \overset{\begin{array}{c} \text{mature epid.,}\\ \text{cort., endo. cells,}\\ \text{all apical cells} \end{array}}{\sum }({\text{Na}}^{+}\text{ fluxes via plasma}\\ \text{ membrane antiporters}); \end{array}$$

(6) $$\begin{array}{l} {\text{cost of Na}}^{+}\text{ transport across stelar cell plasma membranes}=\\ \;\frac{1}{\text{root mass}}\times \overset{\begin{array}{c} \text{mature}\\ \text{stele cells} \end{array}}{\sum }({\text{Na}}^{+}\text{ fluxes via plasma}\\ \text{ membrane antiporters}); \end{array}$$

(7) $$\begin{array}{l} {\text{cost of Na}}^{+}\text{ transport across tonoplasts}=\\ \;\frac{1}{2\times \text{root mass}}\times \overset{\text{all cells}}{\sum }({\text{Na}}^{+}\text{ flux via}\\ \text{ tonoplast antiporters}); \end{array}$$

(8) $$\begin{array}{l} {\text{cost of Cl}}^{-}\text{ transport across outer root cell plasma membranes}=\\ \;\frac{2}{\text{root mass}}\overset{\begin{array}{c} \text{mature epid.,}\\ \text{cort., endo. cells} \end{array}}{\sum }({\text{Cl}}^{-}\text{fluxes via plasma}\\ \text{ membrane symporters}); \end{array}$$

(9) $$\begin{array}{l} {\text{cost of Cl}}^{-}\text{transport across stelar cell plasma membranes}=\\ \;\frac{2}{\text{root mass}}\overset{\begin{array}{c} \text{mature}\\ \text{stele cells} \end{array}}{\sum }({\text{Cl}}^{-}\text{ fluxes via plasma}\\ \text{ membrane symporters}). \end{array}$$

This partitioning of energy requirements into contributions from different root regions, membranes and ion species is more detail than is currently possible to determine by experiment. We also investigated the effect of varying the Na^+^, K^+^, and Ca^2+^ concentrations in the external medium.

To determine the viability of transmembrane ion transport we compared our quantitative estimates of the amount of ATP required to transport ions, based on our model fluxes, with estimates of the total amount of ATP available, based on experimental root respiration rates. *Arabidopsis* root respiration rates measured in the absence of salinity vary from 8 to 30 μmol O_2_ g^−1^ root FW h^−1^ (Álvarez et al., 2012; Alexova et al., 2015; Gravot et al., 2016; Sew et al., 2016). The effect of salinity on the rate of root respiration is unclear as increases, decreases and no change in respiration rate have all been observed to result from exposure to salt stress (Jacoby et al., 2011). Due to this uncertainty, we considered the entire range of root respiration estimates in most of our comparisons. This may be an optimistic estimate of the amount of available energy given that *Arabidopsis* is salt-sensitive, and a similarly salt-sensitive rice (*Oryza sativa* L.) cultivar (IR29) had an approximately 65% reduction in root respiration after 3 weeks of exposure to 25 mM NaCl (Malagoli et al., 2008).

To convert these root respiration rates to the amount of ATP available requires knowledge of the ratio of ATP produced per O_2_ consumed. This ratio depends on the relative contributions of the cytochrome pathway (which provides 5 ATP per O_2_) and the alternative pathway (which provides only 1.75 ATP per O_2_) to the overall respiration rate. Estimates of the proportion of the total respiration flux occurring via the alternative pathway in roots in non-saline conditions are highly variable, ranging from 0 to 60% (Ribas-Carbo et al., 1997; Millar et al., 1998; Millenaar et al., 2000, 2001). In addition, the activity of the alternative pathway is influenced by salt stress (Del-Saz et al., 2016). We assumed a ratio of 4.5 ATP produced per O_2_ (Munns et al., 2020a). Combined with the above measurements of root respiration, this gives 36 to 130 μmol ATP g^−1^ root FW h^−1^. See Figure S1 for a comparison of how this estimate of ATP availability compares with estimates in other studies.

### 2.3. Measuring Salt Tolerance Traits

When identifying transport processes that could be targeted to minimize energy use while achieving desirable responses to salt stress, we assumed the following actions were desirable: maintaining low levels of cytosolic Na^+^; enhancing Na^+^ storage in vacuoles; and controlling the uptake of Na^+^ from the root to the shoot (Munns and Tester, 2008).

## 3. Results

### 3.1. Energy Costs and Their Sources

**Figure 2:**
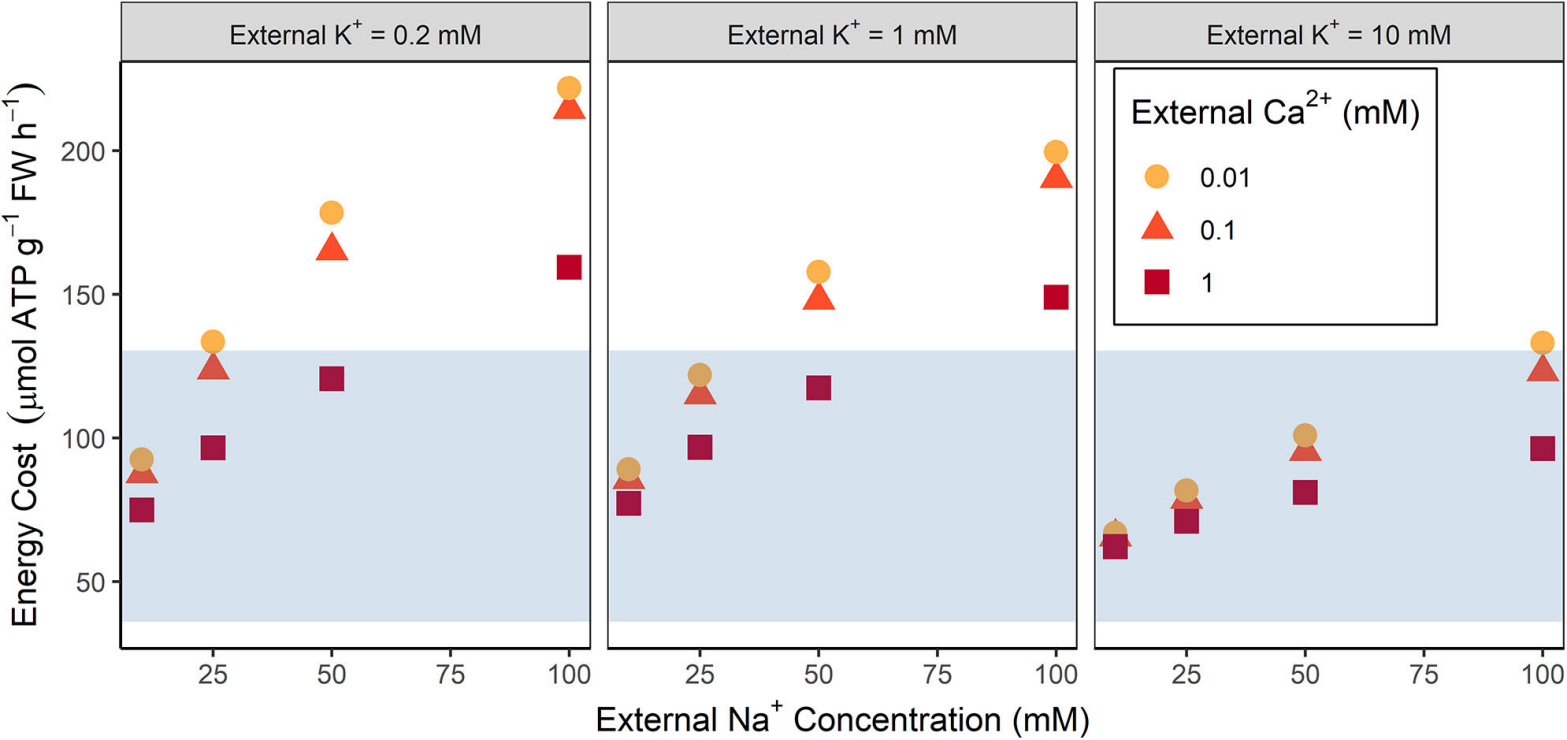
Comparison of the total steady-state energy required for transmembrane ion transport with the energy available from root respiration for a range of external conditions. The blue shading indicates the amount of ATP available based on *Arabidopsis* root respiration rates measured under non-saline conditions (see section 2.2 for details). Energy costs are shown for a range of external Na^+^ concentrations (10, 25, 50, 100 mM), K^+^ concentrations (0.2, 1, 10 mM), and Ca^2+^ concentrations (0.01, 0.1, 1 mM).

The total steady-state energy cost of ion transport increases as the external NaCl concentration increases (see Figure 2). These energy costs are high when compared to the estimates of available energy under stress free conditions (compare symbols and shaded regions in Figure 2), even if an energy surplus is possible. At high external NaCl concentrations the energy demands may exceed the total amount of energy available, while even at low NaCl concentrations the energy demands of just ion transport require a substantial amount of the available ATP.

**Figure 3:**
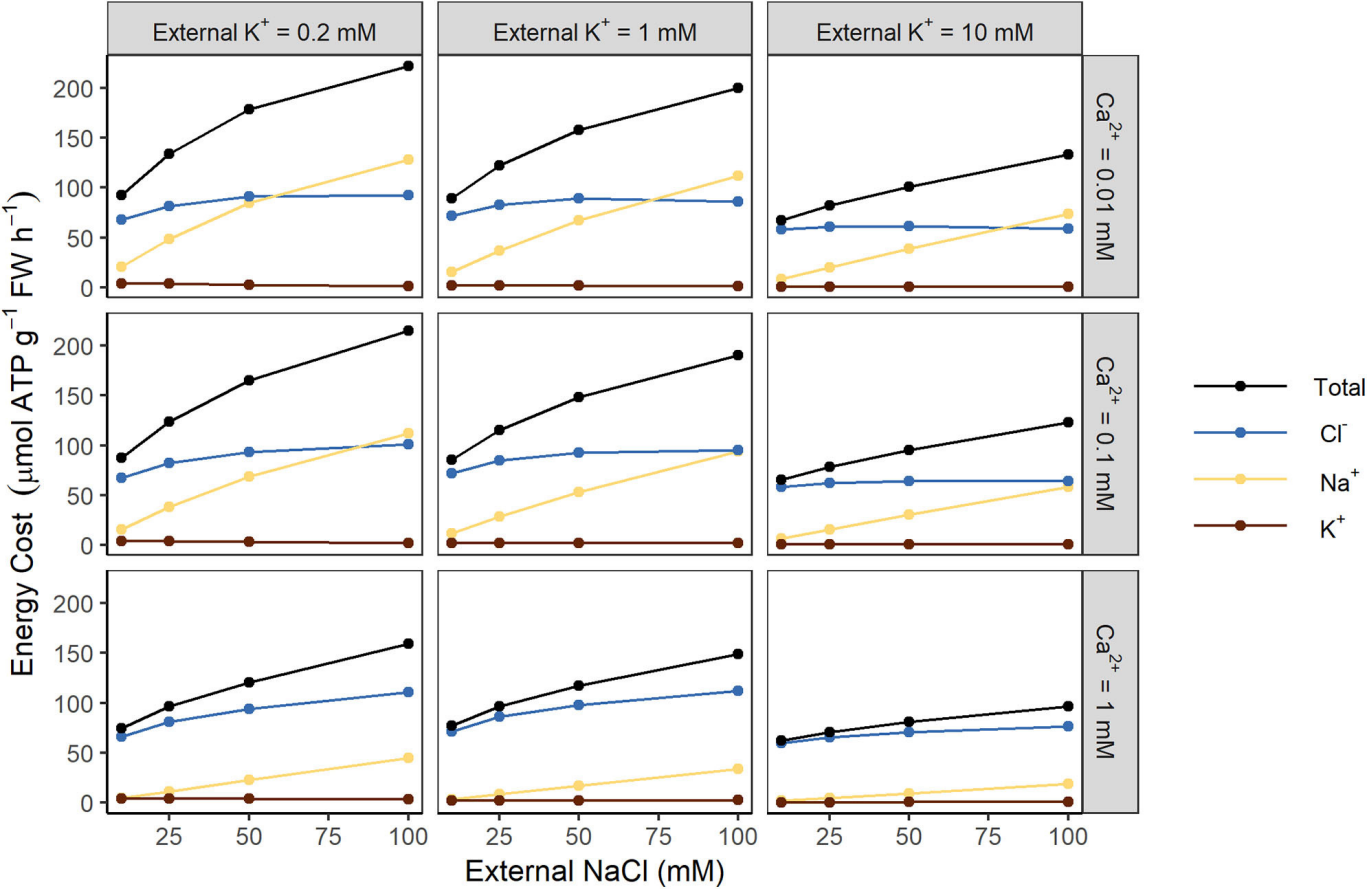
Both Na^+^ and Cl^−^ contribute substantially to the total energy cost of transmembrane ion transport at steady state, while K^+^ does not. Steady-state energy costs are shown for Na^+^ (yellow), Cl^−^ (blue), K^+^ (brown), and the total for all ions (black), for a range of external K^+^ concentrations (0.2, 1, and 10 mM) and external Ca^2+^ concentrations (0.01, 0.1, and 1 mM).

To examine which transport processes place the greatest demand on energy, we quantified the individual energy costs associated with the active transport of each ion (see Figure 3). At high external NaCl concentrations (100 mM), both Na^+^ and Cl^−^ contribute substantially to the overall energy expended. However, at lower external NaCl concentrations the cost of transporting Cl^−^ dominates. In contrast, the cost of transporting K^+^ is negligible (see Figure 3).

**Figure 4:**
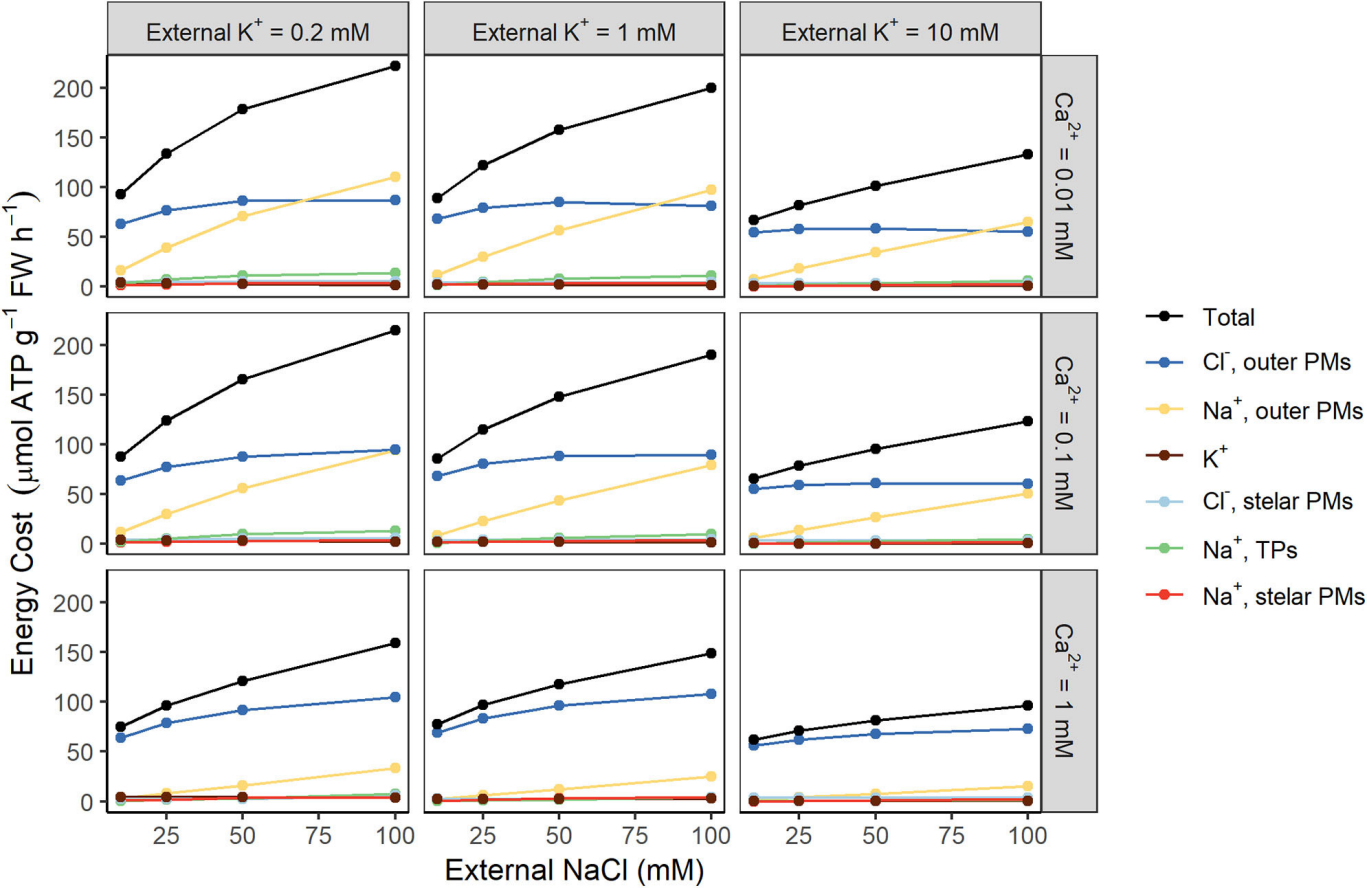
Active efflux of Na^+^ and active uptake of Cl^−^ across the plasma membranes of the outer root cells are the largest contributors to total energy costs. Steady-state energy costs are shown for a range of external K^+^ concentrations (0.2, 1, and 10 mM) and external Ca^2+^ concentrations (0.01, 0.1, and 1 mM). Contributions from the following individual transport processes are shown: Na^+^ transport across the plasma membranes (PMs) of the outer root cells (yellow), the PMs of the stelar cells (red), and the tonoplasts (TPs) of all root tissues (green); Cl^−^ transport across the PMs of the outer root cells (dark blue) and the stelar root cells (light blue); and all transmembrane K^+^ transport (brown). The total cost is shown in black.

To further identify the key sources of energy costs, we investigated the contributions of individual ions to the total energy demand for different root regions (see Figure 4). Active efflux of Na^+^ and active uptake of Cl^−^ across the plasma membranes of the outer root cells place the greatest demand on the available energy (see yellow and dark blue lines, respectively, in Figure 4). In contrast, the upper bound of the energy required to actively store Na^+^ in cell vacuoles is relatively small (green lines), as is the energy expended to actively transport Na^+^ and Cl^−^ across stelar plasma membranes (red and light blue lines).

Figures 2–4 demonstrate that the conditions in the external medium affect the energy costs. For example, energy costs decrease as the external Ca^2+^ concentration increases (see Figure 2) because of reduced energy costs of Na^+^ efflux in the outer root (see Figure 4) resulting from less Na^+^ uptake through NSCCs that have reduced permeability in the presence of higher Ca^2+^ concentrations. Higher external K^+^ concentrations typically lead to lower energy costs (see Figure 2). In agreement with earlier results (Foster and Miklavcic, 2015), lower external K^+^ concentrations lead to more negative trans-plasma membrane potentials. This in turn leads to higher passive Na^+^ influx via channels, leading to higher active Na^+^ efflux and hence higher energy costs.

In addition to the steady state results discussed so far, our model also provides short term energy costs (see Figure S2). These short term results show that energy costs increase rapidly, reaching near steady-state levels in less than 24 h. For high Na^+^ concentrations the amount of available ATP is exceeded only hours after exposure to Na^+^ (see Figure S2).

### 3.2. Minimizing Energy Demand by Altering Outer Root Plasma Membrane Transport Processes

**Figure 5:**
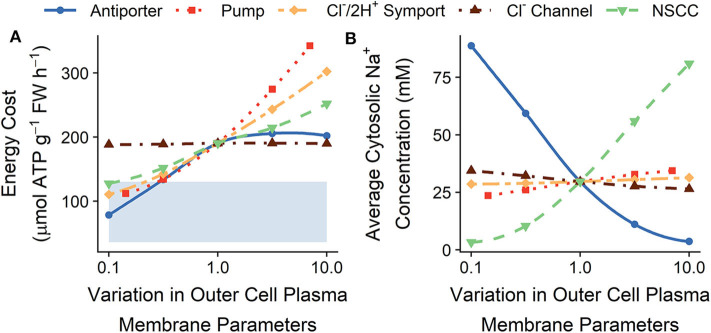
Effect of varying the plasma membrane transport parameters in the outer root cells on the steady-state **(A)** total energy cost of transmembrane ion transport, and **(B)** cytosolic Na^+^ concentration. Line types and colors indicate the plasma membrane parameter varied in the outer root: Na^+^/H^+^ antiporter density (blue, solid line); H^+^ pump density (red, dotted line); Cl^−^/2H^+^ symporter density (yellow, long dot-dashed line); Cl^−^ channel permeability (brown, dot-dashed line); and non-selective cation channel (NSCC) permeability (green, dashed line). The shaded region indicates the total energy available (see section 2 for details). All simulations were conducted with the external medium containing 100 mM NaCl, 1 mM KCl, 0.1 mM Ca^2+^, and having a pH of 5.

Figure 4 indicates that ion transport across the plasma membranes of the outer root cells contributes significantly to the overall energy cost. Active efflux of Na^+^ out of the outer root cells is responsible for maintaining low levels of cytosolic Na^+^ in the root (Foster and Miklavcic, 2019). In this section we identify the transport processes operating on the plasma membranes of outer root cells that can be altered to achieve both lower energy requirements and reasonably low levels of cytosolic Na^+^ (see Figure 5).

Lowering the plasma membrane NSCC permeability and Cl^−^/2H^+^ symporter density in the outer root cells leads to both reduced energy costs (see Figure 5A) and reduced or similar cytosolic Na^+^ concentrations (see Figure 5B). Lowering the NSCC permeability also leads to higher cytosolic K^+^ concentrations, while lowering the Cl^−^/2H^+^ symporter density leads to lower cytosolic Cl^−^ concentrations (see Figure S3).

Increasing the plasma membrane pump and Na^+^/H^+^ antiporter densities would be expected to increase the energy required for transport while reducing the cytosolic Na^+^ concentration. Figure 5A shows that there is an increase in energy costs, although this increase can plateau and can even begin to decrease at high antiporter densities. This occurs because a reduced cytosolic Na^+^ concentration leads to a decreased level of Na^+^ storage in cell vacuoles. Since Na^+^ storage requires energy, this could counteract the increase in energy cost due to increased plasma membrane efflux. Thus, it is possible for increased active efflux out of the outer root cells to lead to decreased cytosolic Na^+^ concentrations without a corresponding increase in energy required (see the plasma membrane Na^+^/H^+^ antiporter density variations in Figure 5). An increase in the H^+^ pump density leads to a slight increase in cytosolic Na^+^ because of charge balance effects. Increasing the pump rate increases active Cl^−^ influx and the concentration of cytosolic buffer anions. This in turn leads to a more negative plasma membrane potential, increasing the passive influx of Na^+^ into root cytosols. This increase in passive influx of Na^+^ exceeds the increase in active Na^+^ efflux resulting from the increased pump density, leading to a slight increase in cytosolic Na^+^. These results highlight the many interacting transport processes occurring in salt-stressed roots.

Changing the K^+^ plasma membrane transport parameters (including the IRC and ORC permeabilities and K^+^/H^+^ symporter density) does not significantly affect energy usage (results not shown), which is unsurprising given the small contribution of K^+^ transport to the overall energy costs (see Figure 3).

Passive Na^+^ uptake as well as active Cl^−^ uptake across the outer root cell plasma membranes are important transport mechanisms to target in order to maintain physiological levels of cytosolic Na^+^ at a lower energy cost. Once Na^+^ has entered the symplast, its removal requires active transport and hence energy. Therefore, preventing Na^+^ from entering the symplast in the first place by maintaining a low NSCC permeability in the outer root cells significantly reduces the energy cost of Na^+^ exclusion.

### 3.3. Minimizing Energy Demand by Altering Stelar Plasma Membrane Transport Processes

**Figure 6:**
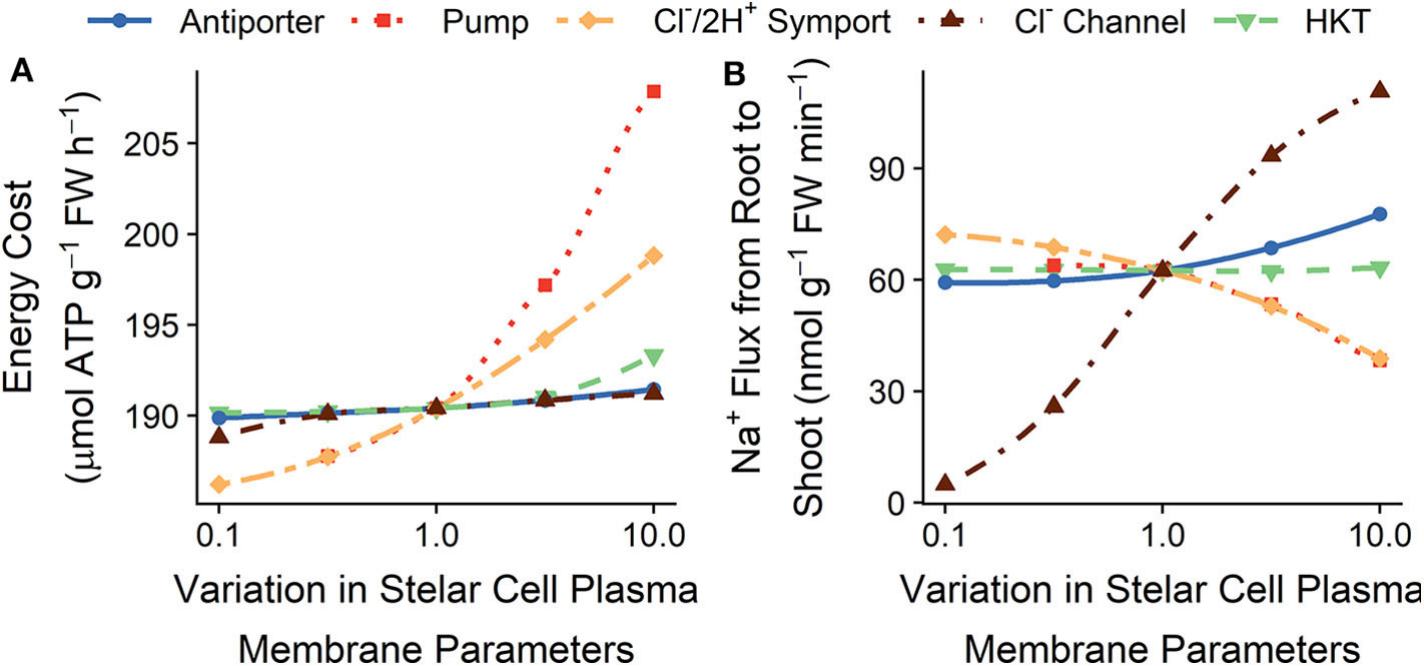
Effect of varying the plasma membrane transport parameters in the stelar root cells on the steady-state **(A)** total energy cost of transmembrane ion transport, and **(B)** Na^+^ flux from the root to the shoot. Line types and colors indicate the plasma membrane parameter varied in the stelar cells: Na^+^/H^+^ antiporter density (blue, solid line); H^+^ pump density (red, dotted line); Cl^−^/2H^+^ symporter density (yellow, long dot-dashed line); and Cl^−^ channel permeability (brown, dot-dashed line); and HKT permeability (green, dashed line). Note the changes in energy costs are so small that, for visual clarity, a shaded region indicating the total energy available is not included. All simulations were conducted with the external medium containing 100 mM NaCl, 1 mM KCl, 0.1 mM Ca^2+^, and having a pH of 5.

Figure 6 shows the effects of varying stelar plasma membrane transport parameters on the energy costs of ion transport and the rate of Na^+^ transport from the root to the shoot. We have previously demonstrated that Na^+^ loading into the xylem transpiration stream is an active process, while unloading is a passive process (Foster and Miklavcic, 2019). Consequently, reducing the active transport of Na^+^ into the transpiration stream (by reducing the stelar plasma membrane Na^+^/H^+^ antiporter densities) leads to both reduced energy expended (see Figure 6A) and reduced Na^+^ flux from the root to the shoot (see Figure 6B), although for these simulation conditions the effects are minor. However, due to the influence of the H^+^ pump on Cl^−^ transport, changes to the H^+^ pump density do not always lead to the expected changes in Na^+^ flux (see Figure 6B).

Increasing the passive Na^+^ permeability of stelar cells (by increasing the HKT1;1 permeability) increases the passive unloading of Na^+^ from the xylem transpiration stream, which in turn leads to an increase in active loading of Na^+^ into the xylem, increasing the futile cycling of Na^+^ across the stelar plasma membranes. Hence, energy costs increase (see green dashed lines in Figure 6A) without an associated decrease in Na^+^ flux to the shoot (see green dashed lines in Figure 6B).

In contrast to Na^+^, Cl^−^ is passively loaded into the xylem transpiration stream and is actively unloaded. Therefore, lowering the stelar plasma membrane Cl^−^ channel permeability leads to lower Cl^−^ flux from the root to the shoot (see Figure S4), as well as lower energy costs because of less futile cycling of Cl^−^ across the stelar plasma membranes (see Figure 6A). To maintain charge balance, the reduction in Cl^−^ flux also leads to a lower Na^+^ flux from the root to the shoot (see Figure 6B). The converse is true upon a lowering of the Cl^−^/2H^+^ symporter density.

Varying the plasma membrane K^+^ transport parameters in the stelar cells does not substantially affect the steady-state energy demands (results not shown).

The changes in energy requirements resulting from altered plasma membrane transport in stelar cells are much smaller than the changes possible by altering plasma membrane transport in the outer root cells. This is a reflection of the initial influx of Na^+^ and Cl^−^ into the root being the major contributor to the overall energy required for ion transport.

### 3.4. Minimizing Energy Demand by Altering Tonoplast Transport Processes

**Figure 7:**
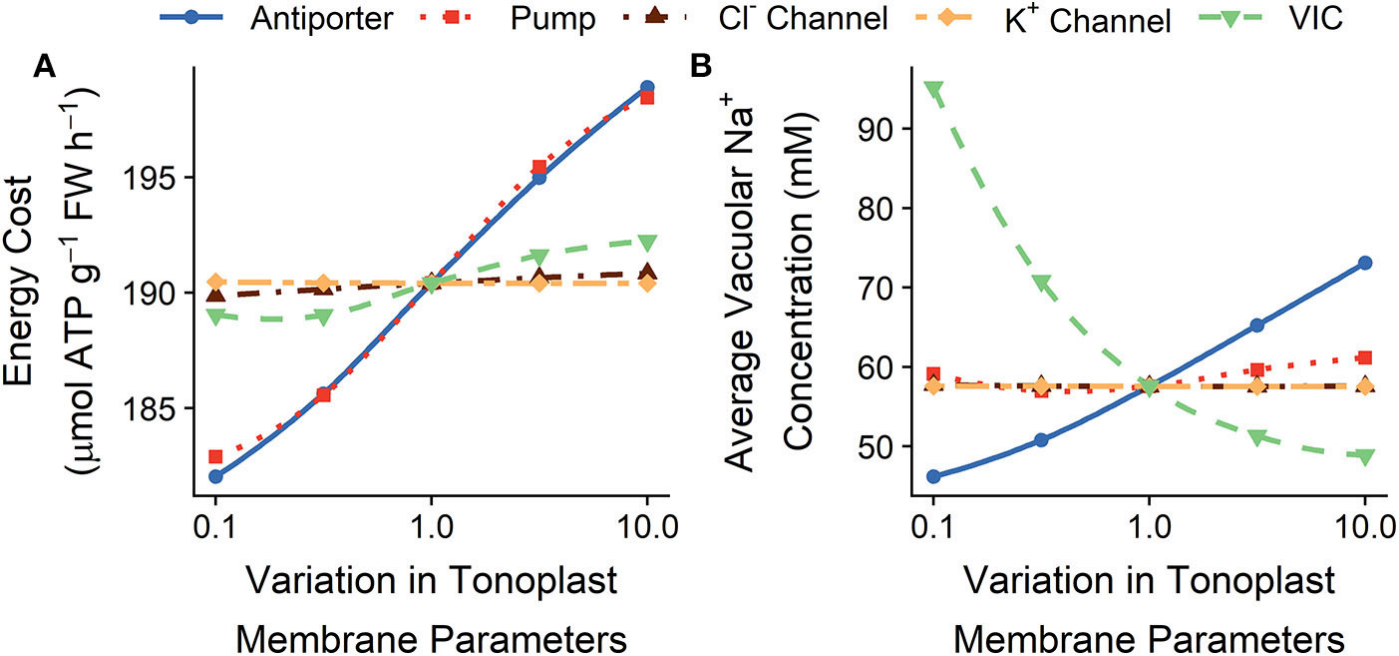
Effect of varying the tonoplast transport parameters in all root cells on the steady-state **(A)** total energy cost of transmembrane ion transport, and **(B)** average vacuolar Na^+^ concentrations. Line types and colors indicate the tonoplast parameter varied: Na^+^/H^+^ antiporter density (blue, solid line); H^+^ pump density (red, dotted line); Cl^−^ channel permeability (brown, dot-dashed line); K^+^ channel permeability (yellow, long dot-dashed line); and voltage insensitive channel permeability (green, dashed line). Note the changes in energy costs are so small that, for visual clarity, a shaded region indicating the total energy available is not included. All simulations were conducted with the external medium containing 100 mM NaCl, 1 mM KCl, 0.1 mM Ca^2+^, and having a pH of 5.

Figure 7 shows the effects of varying the tonoplast transport parameters on the total energy required to transport ions and on the amount of Na^+^ stored in all root cell vacuoles. Lowering the tonoplast passive Na^+^ permeability increases the amount of Na^+^ stored in root cell vacuoles, while simultaneously decreasing the amount of energy required to maintain this storage (see green lines in Figure 7). Increasing the active influx of Na^+^ into the vacuoles also typically increases the amount of Na^+^ stored, but this requires more energy (see red and blue lines in Figure 7). Varying the K^+^ and Cl^−^ tonoplast channel permeabilities does not substantially affect the steady-state energy requirements or the concentration of ions stored in vacuoles.

While varying the tonoplast transport parameters can significantly affect the steady-state vacuolar Na^+^ concentrations (see Figure 7B), this variation does not lead to significant changes in steady-state cytosolic ion concentrations or ion fluxes from the root to the shoot (see Figure S5).

**Figure 8:**
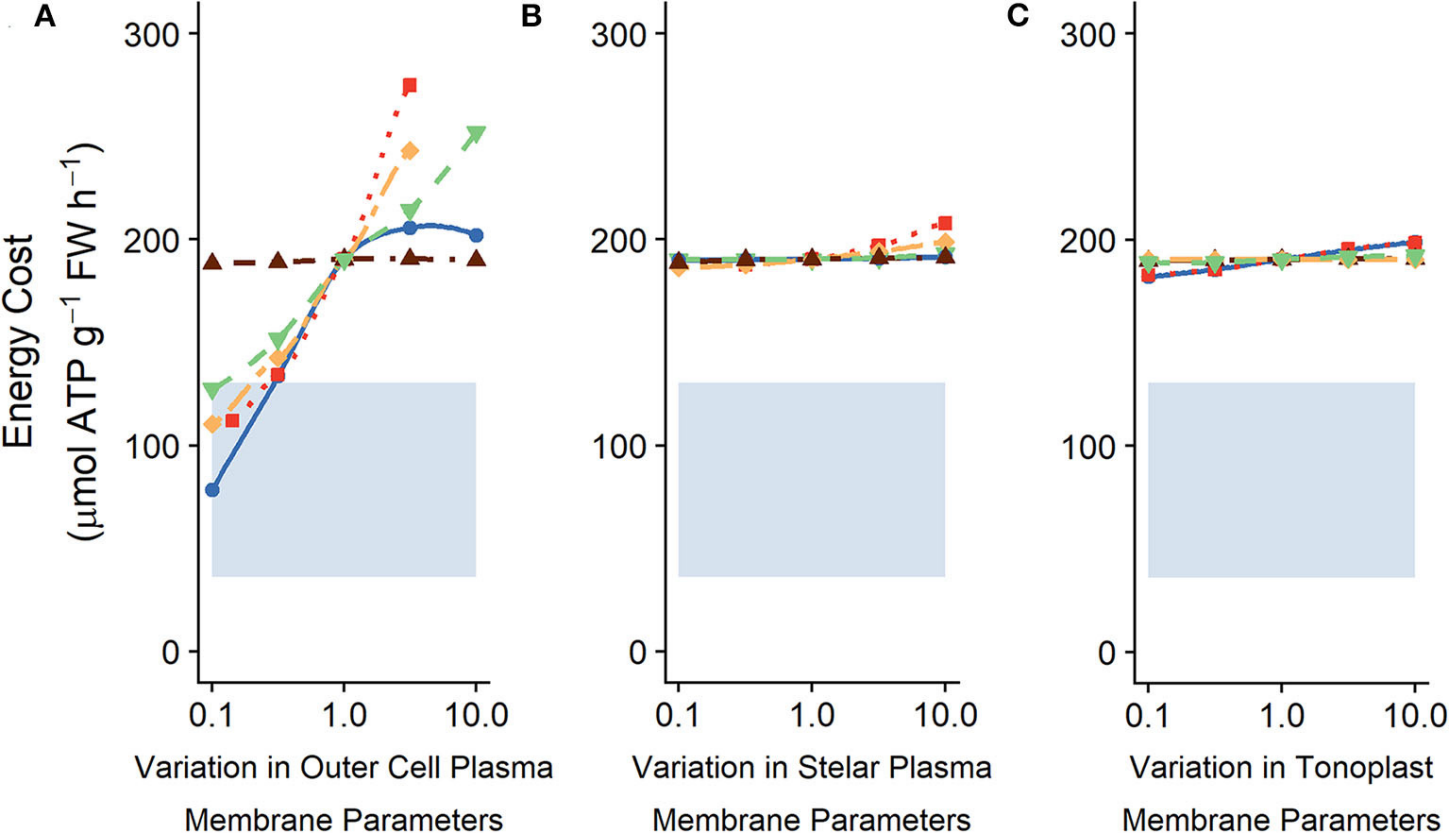
Comparing effects of varying **(A)** plasma membrane parameters in the outer root cells, **(B)** plasma membrane parameters in the stele and **(C)** tonoplast transport parameters in all root cells on the steady-state energy costs. The shaded region indicates the total energy available (see section 2 for details). The model results shown here are the same as those shown in Figures 5A, 6A, 7A, but displayed with the same scale for easier comparison. In (A) line types and colors indicate the plasma membrane parameter varied in the outer root: Na^+^/H^+^ antiporter density (blue, solid line); H^+^ pump density (red, dotted line); Cl^−^/2H^+^ symporter density (yellow, long dot-dashed line); Cl^−^ channel permeability (brown, dot-dashed line); and voltage insensitive channel permeability (green, dashed line). In **(B)** line types and colors indicate the plasma membrane parameter varied in the stelar cells: Na^+^/H^+^ antiporter density (blue, solid line); H^+^ pump density (red, dotted line); Cl^−^/2H^+^ symporter density (yellow, long dot-dashed line); and Cl^−^ channel permeability (brown, dot-dashed line); and HKT permeability (green, dashed line). In **(C)** line types and colors indicate the tonoplast parameter varied: Na^+^/H^+^ antiporter density (blue, solid line); H^+^ pump density (red, dotted line); Cl^−^ channel permeability (brown, dot-dashed line); K^+^ channel permeability (yellow, long dot-dashed line); and voltage insensitive channel permeability (green, dashed line). All simulations were conducted with the external medium containing 100 mM NaCl, 1 mM KCl, 0.1 mM Ca^2+^, and having a pH of 5.

The change in the energy expended from altering the tonoplast transport parameters is small in comparison with the change that can be achieved by altering the plasma membrane transporter strengths in the outer root (compare Figures 8A and 8C). This difference is similar to the energy difference observed upon varying plasma membrane transporters in the outer root cells compared with transporters in stellar cells (Figures 8A,B).

## 4. Discussion

### 4.1. The Energy Required for Transport in Salt-Stressed Roots May Exceed the Energy Available

Using our detailed biophysical model we found that the energy required for transmembrane ion transport in salt-stressed roots at steady-state is similar in magnitude to, or exceeds, the estimated total energy supply through root respiration, especially for high external NaCl conditions. This was despite our energy expenditure estimates not including the energy required for growth, repair and maintenance, or any unknown energy requirements for active transport across cell membranes, such as across mitochondrial membranes. However, given the uncertainty about the exact amount of ATP available in roots (Munns et al., 2020a), it may be premature to unequivocally conclude that the energy demand exceeds energy supply. If nothing else, our conservative calculations indicate a need to obtain more accurate experimental estimates of energy production from root respiration and H^+^:ATP stoichiometry especially under salt stress conditions. Despite this uncertainty, our analysis suggests that the energy requirements for ion transport under high external NaCl conditions are a significant factor in the salt stress response.

Since our model is based on currently understood and accepted ion transport mechanisms, our predictions suggest there is a need to re-assess these mechanisms. Based on our cost estimates, to achieve realistic levels of energy demand, the secondary active transport of Na^+^ and Cl^−^ across outer root cell plasma membranes would need to be more energy efficient and/or the energy source for primary active transport across plasma membranes would need to use less ATP. There is currently no evidence for the existence of Na^+^ transporters capable of actively effluxing Na^+^ out of the root that are more energy efficient than Na^+^/H^+^ antiporters. Cation-chloride cotransporters (CCCs) have been suggested as a possibility (Britto and Kronzucker, 2009). However, it has been shown that the CCCs thus far identified are either localized to the Golgi and the trans-Golgi network, not plasma membranes (Henderson et al., 2015), or do not transport Na^+^ (Kong et al., 2011), precluding a role in the Na^+^ efflux out of the outer root. More energy efficient Cl^−^ transport than that assumed here could occur if the plasma membrane Cl^−^/2H^+^ symporter densities were lower than those assumed in our model.

The energy demands for all ion transporters would be lower than those calculated here if the H^+^ pump stoichiometries differed from the assumed values. The H^+^/ATP coupling ratio of the plasma membrane H^+^-ATPase has been shown to increase under salt stress in cucumber roots (Janicka-Russak et al., 2013) and in *Sorghum bicolor* roots (Miranda et al., 2017), as well as under osmotic stress in tomato cells (Kerkeb et al., 2002). For example, Janicka-Russak et al. (2013) calculated a coupling ratio of approximately three H^+^ to one ATP for the plasma membrane H^+^-ATPase after 6 days of treatment with 50 mM NaCl. This could reduce the total energy demand for plasma membrane ion transport by one-third of that required for the commonly assumed coupling ratio of one H^+^ to one ATP, making the energy costs of ion transport during salt stress feasible. The possibility that the plasma membrane H^+^-ATPase coupling ratio increases under salt stress could be an important factor in salt tolerance and is worthy of further investigation.

Our model results add weight to another possibility for more energy efficient ion transport. We have assumed that active proton efflux across the plasma membranes occurs via proton pumps. However, Wegner and Shabala (2019) have recently proposed that the pH gradient (low in the apoplast, high in the cytosol) which drives the Na^+^/H^+^ antiporter may alternatively or additionally be maintained by means of an active buffering mechanism (called a “biochemical pH clamp” by the authors): protons are generated in the apoplast and “scavenged” in the cytosol by respective metabolic processes. This hypothesis would alleviate the energy demand on active transport by pump activity alone. The proposal is intriguing and is worth exploring both experimentally and theoretically, perhaps initially at the level of a single cell (Foster and Miklavcic, 2015) in order to establish the required model parameter values.

### 4.2. Na^+^ and Cl^−^ Transport Across Outer Root Plasma Membranes Are the Largest Contributors to Energy Costs

We identified active Na^+^ efflux and Cl^−^ uptake across the outer root plasma membranes as the major energy consumers involved in transport under conditions of high external NaCl. This suggests that energy cost calculations based on tracer fluxes across outer root plasma membranes could provide a reasonable estimate of the total energy cost of transport, with only a small underestimation (7 to 13% lower than the actual total energy cost, using the simulations shown in Figure 2). These fluxes are more feasible to measure experimentally than fluxes across tonoplast or stelar plasma membranes. However, since the cost of transporting Cl^−^ is significant, Cl^−^ fluxes should also be measured in addition to Na^+^ fluxes.

**Figure 9:**
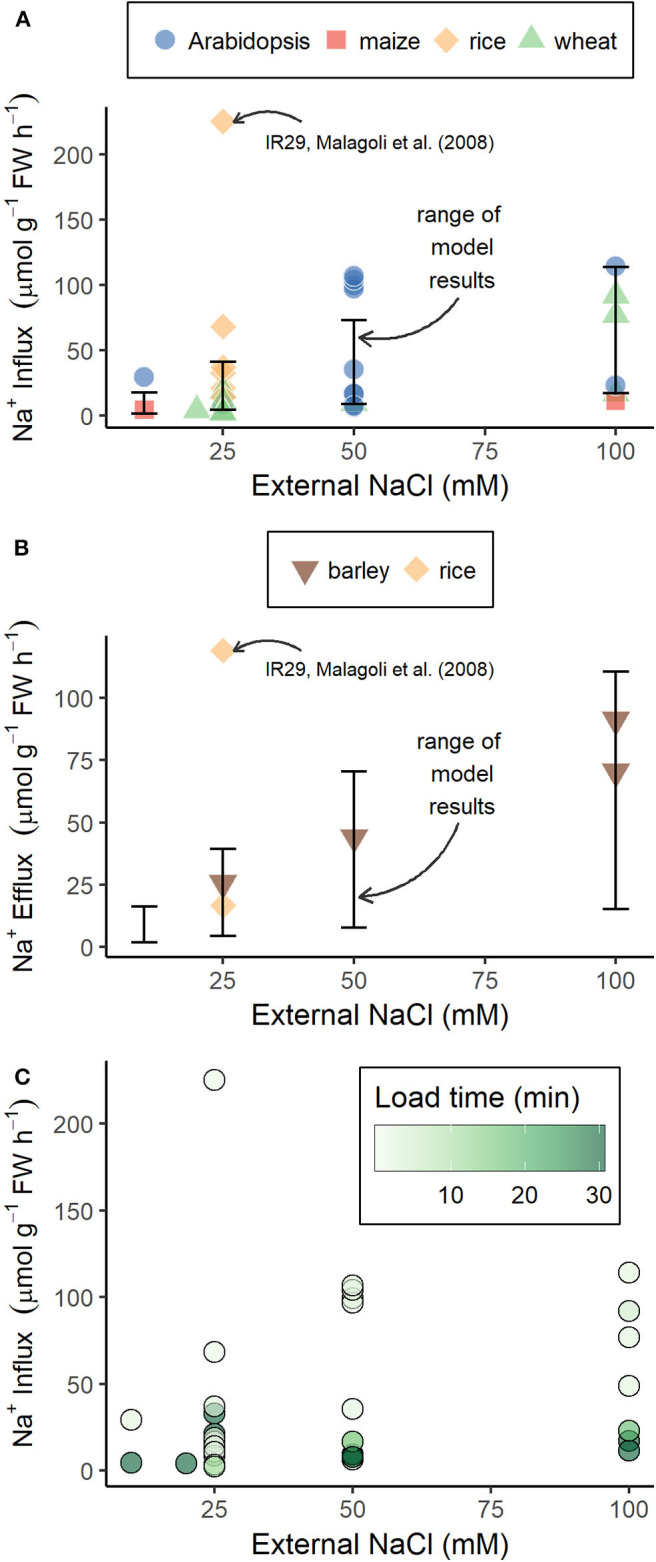
Model steady-state Na^+^ influxes and effluxes across outer root cell plasma membranes are similar in magnitude to influxes and effluxes measured using radioactive tracers. **(A)** Na^+^ influxes measured using ^22^Na^+^ or ^24^Na^+^ (symbols), or simulated using our model (error bars); **(B)** Na^+^ effluxes measured using ^24^Na^+^ (symbols) or simulated using our model (error bars); and **(C)** experimental Na^+^ influxes shown in **(A)** with tracer loading time indicated by color. Experimental results were obtained from Malagoli et al. (2008), Essah et al. (2003), Wang et al. (2006), Wang et al. (2009), Maathuis and Sanders (2001), Elphick et al. (2001), Moller et al. (2009), Jha et al. (2010), Davenport and Tester (2000), Laurie et al. (2002), Zidan et al. (1991), Davenport et al. (2005), Davenport et al. (2007), Demidchik et al. (2004), Flam-Shepherd et al. (2018), Kronzucker et al. (2006), and Kronzucker et al. (2008). Error bars indicate the range of model Na^+^ fluxes across outer root cell plasma membranes obtained for the range of external conditions used in the simulations in Figure 2.

The use of tracer fluxes to estimate total energy demand assumes that these fluxes accurately reflect trans-plasma membrane fluxes. However, it has been suggested that the mismatch between available and required energy, estimated using Na^+^ tracer fluxes, could be due to these tracer fluxes not accurately reflecting the trans-plasma membrane Na^+^ fluxes (Britto and Kronzucker, 2015; Flam-Shepherd et al., 2018). Our model trans-plasma membrane Na^+^ influxes and effluxes are similar in magnitude to the majority of the Na^+^ influxes (see Figure 9A) and effluxes (see Figure 9B) measured using radioactive tracers. The simulated and experimentally measured Na^+^ fluxes cover a wide range of values. This is in part due to the effects of different external medium conditions, as demonstrated by the wide range of model results resulting from the range of external medium conditions simulated. However, the experimentally measured fluxes are also affected by the choice of experimental protocol. For example, the tracer loading time substantially affects Na^+^ influx measurements, with shorter loading times resulting in higher fluxes (see Figure 9C). This effect of loading time is important because it is not clear whether higher fluxes measured using shorter loading times, or lower fluxes measured using longer loading times, accurately reflect trans-plasma membrane fluxes. Influx measurements taken using longer loading times are affected by tracer effluxes, which increase over time, leading to lower apparent influxes (Britto and Kronzucker, 2001). However, influxes measured using shorter loading times may be affected by initial rapid diffusion of the tracer that is not representative of the fluxes of the bulk Na^+^ (Munns et al., 2020b). Or a combination of these processes could be occurring, making interpretation of the fluxes over any time frame difficult. It has also been proposed that tracer influxes primarily constitute fluxes rapidly cycling through the apoplast without crossing any membranes and thus not consuming any energy (Britto and Kronzucker, 2015; Flam-Shepherd et al., 2018). However, it is unclear what forces would drive this proposed recirculation of Na^+^ through the root apoplast. If influx of the tracer into the apoplast is a passive process, efflux of the tracer would require active transport. A clearer understanding of what tracer fluxes are actually measuring is required for them to be useful for understanding energy costs, or other aspects of salt tolerance. This clearer understanding could be achieved in the future by adding a specific Na^+^ tracer species to our model.

Active uptake of Cl^−^ via the Cl^−^/2H^+^ symporter in the outer root cells is energetically expensive, given that two ATP are required per Cl^−^ transported. However, it is possible that these energy estimates are biased by the fact that Cl^−^ is the only mobile anion in our model. On the other hand, the results are consistent with the high energy costs of Cl^−^ uptake estimated for barley roots (Teakle and Tyerman, 2010). In addition, the model pre-salt energy cost of trans-plasma membrane ion transport (consisting almost exclusively of the cost of transporting Cl^−^) is 44 μmol ATP g^−1^ root FW h^−1^, which is between 34 and 122% of the available energy (assuming 1 mM KCl, 0.1 mM Ca^2+^ and a pH of 5 in the external medium). These pre-salt energy values are a similar order of magnitude to, although higher than, previous estimates of energy requirements for nitrate uptake under unstressed conditions (10–70% of the energy available from root respiration, Kurimoto et al., 2004). The model Cl^−^ transporter strengths could be estimated with more certainty in the future through further comparisons with experiment.

### 4.3. Minimizing Energy Requirements

Reducing the passive leakage of Na^+^ across tonoplasts and outer root plasma membranes provides a win-win scenario of lower energy consumed as well as greater Na^+^ storage and greater Na^+^ exclusion, suggesting that these channels could be important targets for improved salt tolerance. Interestingly, *Thellungiella halophila* (a halophyte relative of *Arabidopsis*) has been identified as having lower Na^+^ influx than *Arabidopsis* (Wang et al., 2006). Unfortunately, the genetic identity of the channels responsible for passive Na^+^ transport into the outer root cells and across tonoplasts remain unknown (Munns et al., 2020a).

It is puzzling that several stages of Na^+^ transport across roots appear to be very inefficient in terms of energy use. In particular, why do outer root cells have such high Na^+^ influxes? A possible explanation is that these high Na^+^ influxes are necessary to allow for initial osmotic adjustment in the root and shoot and hence assist with water uptake. In addition, coupled cotransport of water and ions, including Na^+^, has been suggested to occur across the plasma membranes of xylem parenchyma cells (Wegner, 2017). Similar cotransport of water and Na^+^ across the plasma membranes of outer root cells could potentially lead to beneficial enhancements in water transport. The aquaporin AtPIP2;1, which is highly expressed in root epidermal cell plasma membranes and is a candidate for the NSCC responsible for Na^+^ uptake by roots, is permeable to both water and Na^+^ when expressed in *Xenopus laevis* oocytes (Byrt et al., 2017). However, direct coupling of water and Na^+^ transport has not yet been demonstrated. Another conundrum is why plant roots use energy to actively load Na^+^ into the xylem transpiration stream. Our previous modeling suggests that this active loading of Na^+^ enhances the amount of water taken up by the root (Foster and Miklavcic, 2019).

Reducing the amount of active Cl^−^ uptake into the outer root cells could also reduce energy costs. However, if the relevant transporters and channels have low selectivity for Cl^−^ and ${\text{NO}}_{3}^{-}$, it is possible that these changes could negatively impact on ${\text{NO}}_{3}^{-}$ uptake under unstressed conditions. Unfortunately, to date only a single gene responsible for trans-plasma membrane Cl^−^ transport in the outer root has been identified (Li et al., 2017a,b).

### 4.4. Conclusions and Future Directions

Through our application of a comprehensive model of ion transport greater light has been shed on the energy requirements of the individual ion transport mechanisms operating in salt-stressed roots. Our improved understanding of the relative importance of different mechanisms points to ways to improve the salt tolerance of food crops. Our calculated high energy costs of transmembrane ion transport suggest that breeders and geneticists should investigate the effects of altering passive Na^+^ leakage and active Cl^−^ uptake across outer root plasma membranes as potential targets for improved energy efficiency.

Given the uncertainty surrounding estimates of the amount of ATP provided by root respiration it is unclear whether the energy requirements of ion transport in salt-stressed roots exceed energy supply. However, on the basis of existing knowledge it would appear that an energy deficit is the case. The uncertainty surrounding the energy supply could be addressed in the future by incorporating mitochondria into our model, allowing both energy supply and demand to be simulated. This could be achieved first at the single cell level (e.g., Foster and Miklavcic, 2015) and then expanded to organ and whole plant models. Comparisons between such model predictions and experimental measurements would provide greater clarity on the feasibility of ion transport mechanisms. In addition, a combination of experimentation and modeling could be used to explore the accuracy of radioactive tracer flux measurements, as well as the possibility of a variable H^+^/ATP coupling ratio for the plasma membrane H^+^-ATPase.

## Data Availability Statement

All datasets generated for this study are included in the article/Supplementary Material.

## Author Contributions

The authors contributed equally to the research design, the interpretation of results and the writing of the manuscript. KF performed the simulations and produced the figures.

## Conflict of Interest

The authors declare that the research was conducted in the absence of any commercial or financial relationships that could be construed as a potential conflict of interest.

## Footnotes

^1^Available at https://www.frontiersin.org/articles/10.3389/fpls.2019.01121/full#supplementary-material.
