# Supplementary material for: A Comprehensive Biophysical Model of Ion and Water Transport in Plant Roots. III. Quantifying the Energy Costs of Ion Transport in Salt-Stressed Roots of Arabidopsis
Source: Front Plant Sci. 2020 Jul 3;11:865. doi: 10.3389/fpls.2020.00865 (PMC7348042; doi:10.3389/fpls.2020.00865)
Supplement: Supplementary file 1 [file Data_Sheet_1.PDF]

Supplementary Material

1 ADDITIONAL RESULTS

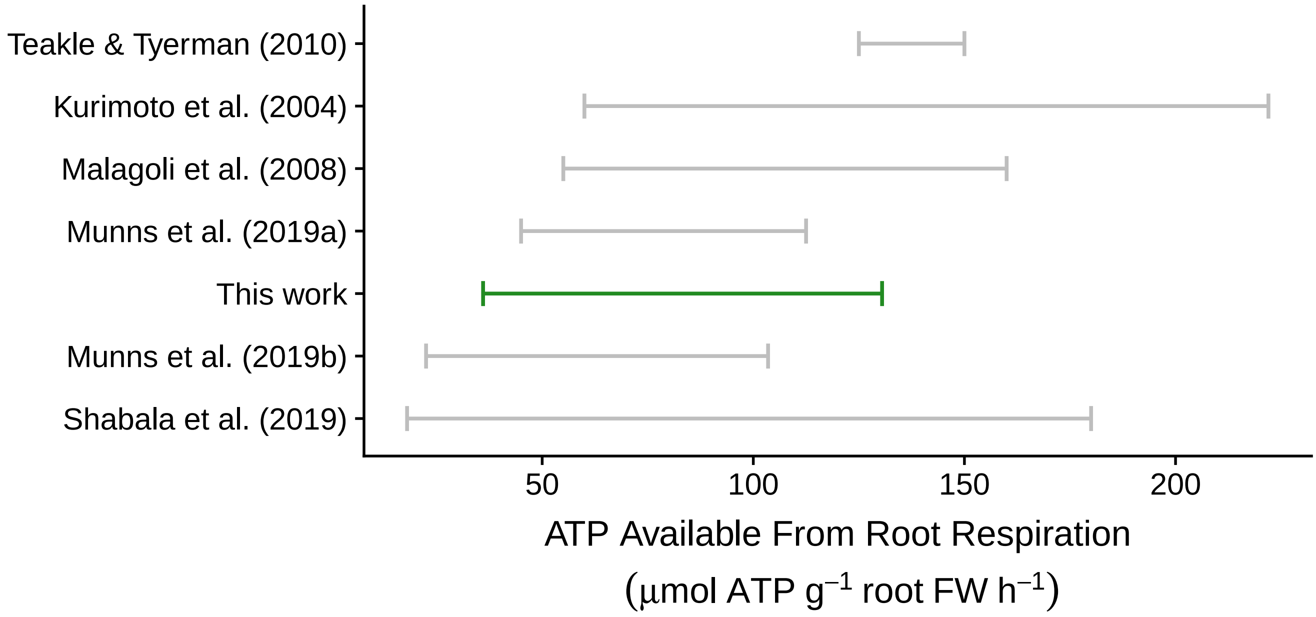

Figure S1. Comparison of estimates of the amount of ATP available from root respiration used in this work (shown in green) and in the literature (Teakle and Tyerman, 2010; Kurimoto et al., 2004; Malagoli et al., 2008; Munns et al., 2019a,b; Shabala et al., 2019).

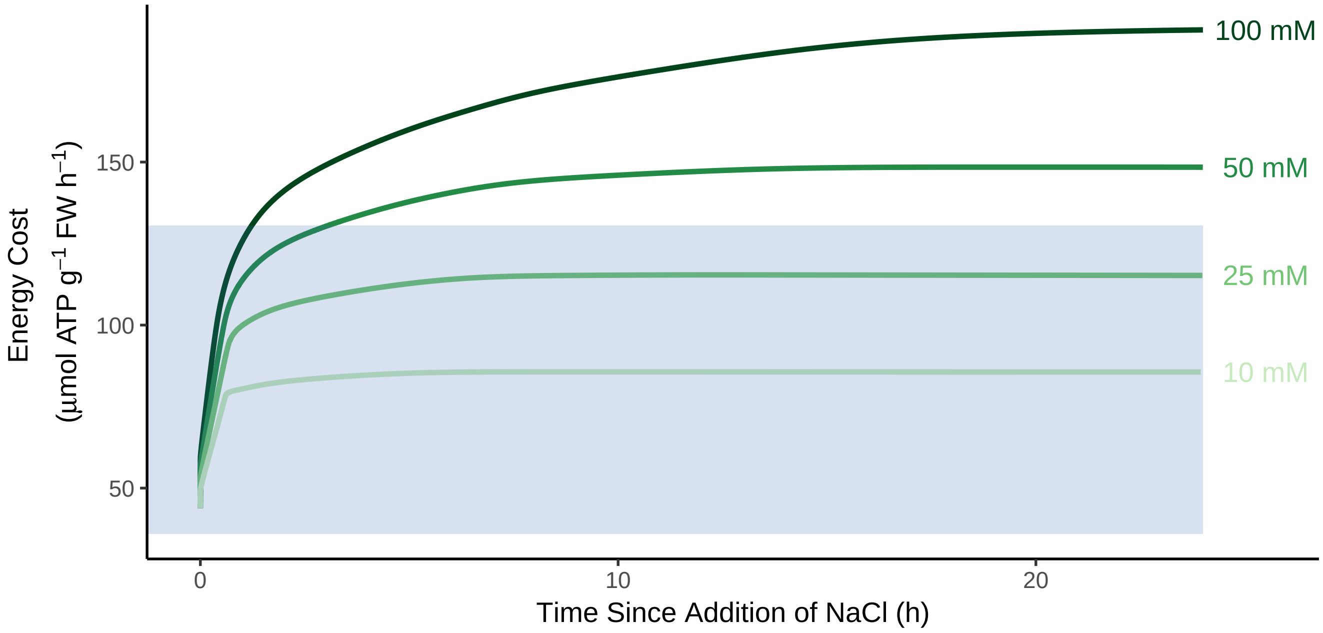

Figure S2. Short term increases in energy costs after exposure to 100 mM, 50 mM, 25 mM and 10 mM NaCl. The blue shading indicates the amount of ATP available based on *Arabidopsis* root respiration rates measured under non-saline conditions. All simulations were conducted with the external medium containing 1 mM KCl, 0.1 mM Ca^{2+} and having a pH of 5.

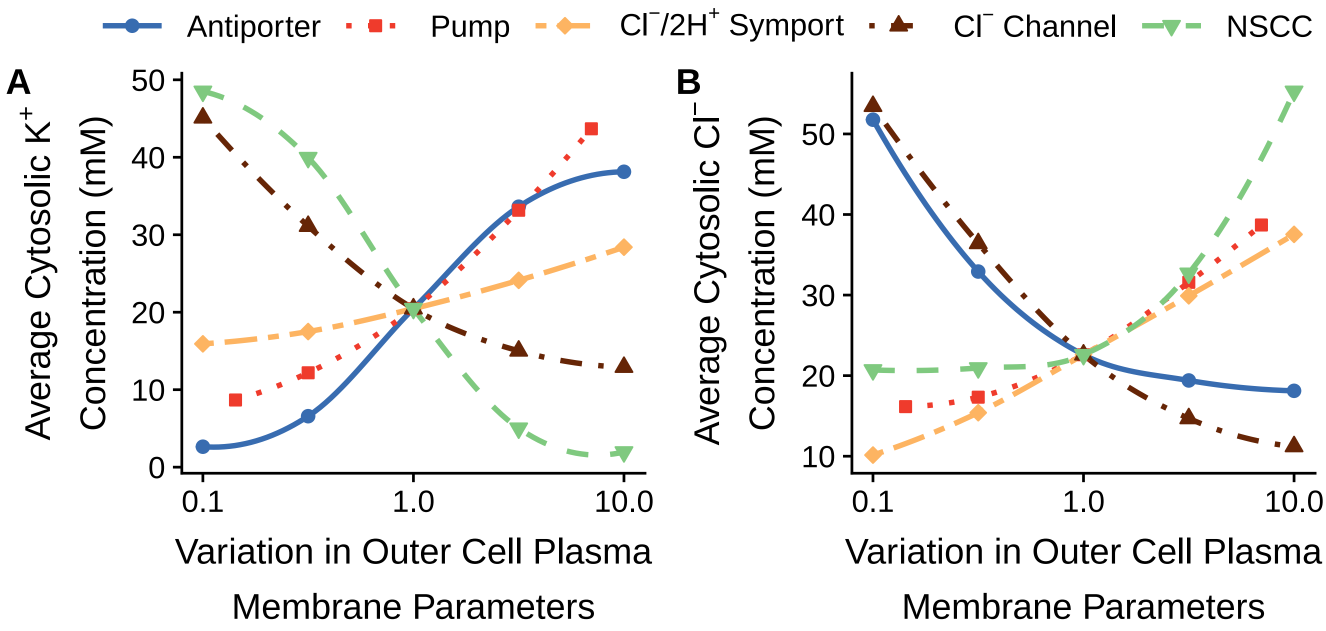

Figure S3. Effect of varying the plasma membrane transport parameters in the *outer root cells* on the steady-state average cytosolic **(A)** K^+ and **(B)** Cl^- concentrations. Line types and colours indicate the plasma membrane parameter varied in the outer root: Na^+/H^+ antiporter density (blue, solid line); H^+ pump density (red, dotted line); $Cl^-/2H^+$ symporter density (yellow, long dot-dashed line); Cl^- channel permeability (brown, dot-dashed line); and nonselective cation channel (NSCC) permeability (green, dashed line). All simulations were conducted with the external medium containing 100 mM NaCl, 1 mM KCl, 0.1 mM Ca^{2+} and having a pH of 5.

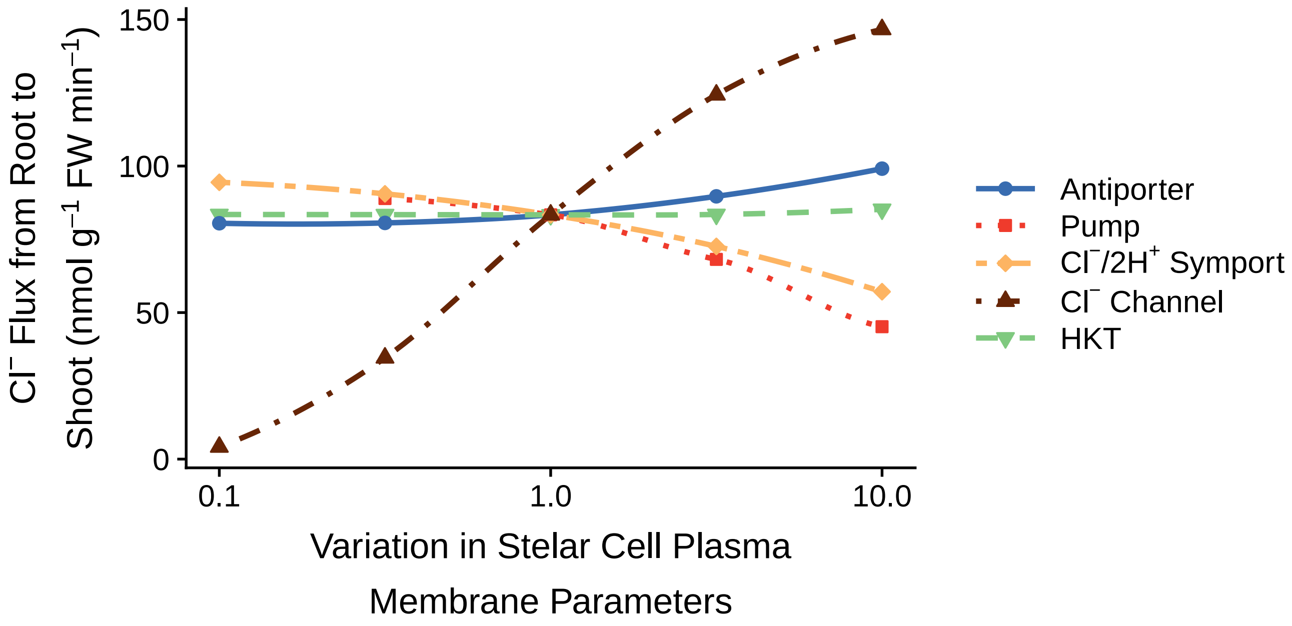

Figure S4. Effect of varying the plasma membrane transport parameters in the *stelar root cells* on the steady-state Cl^- flux from root to shoot. Line types and colours indicate the plasma membrane parameter varied in the stelar cells: Na^+/H^+ antiporter density (blue, solid line); H^+ pump density (red, dotted line); $Cl^-/2H^+$ symporter density (yellow, long dot-dashed line); Cl^- channel permeability (brown, dot-dashed line); and HKT permeability (green, dashed line). All simulations were conducted with the external medium containing 100 mM NaCl, 1 mM KCl, 0.1 mM Ca^{2+} and having a pH of 5.

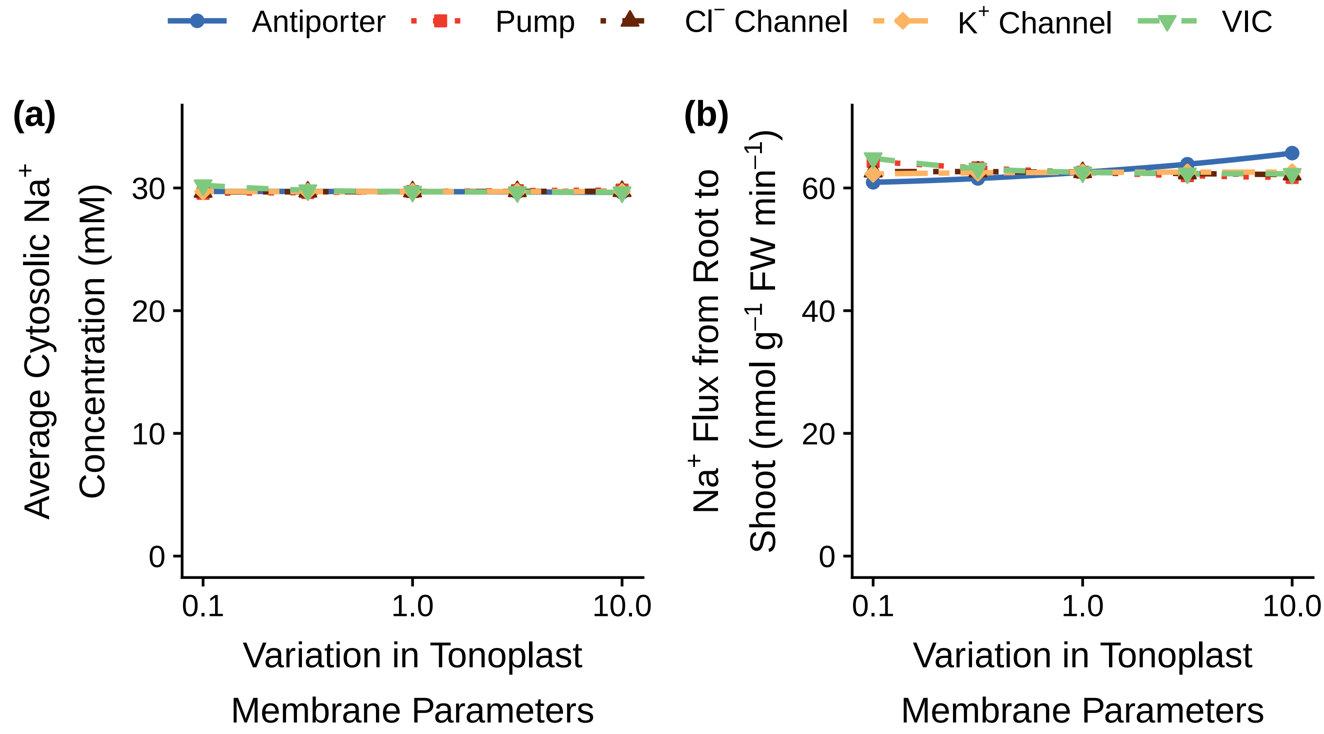

Figure S5. Effect of varying the *tonoplast* transport parameters in all root cells on the steady-state **(A)** average cytosolic Na^+ concentrations and **(B)** Na^+ flux from the root to the shoot. Line types and colours indicate the tonoplast parameter being varied: Na^+/H^+ antiporter density (blue, solid line); H^+ pump density (red, dotted line); Cl^- channel permeability (brown, dot-dashed line); K^+ channel permeability (yellow, long dot-dashed line); and voltage insensitive channel permeability (green, dashed line). All simulations were conducted with the external medium containing 100 mM NaCl, 1 mM KCl, 0.1 mM Ca^{2+} and having a pH of 5.

REFERENCES

Kurimoto, K., Day, D.A., Lambers, H., Noguchi, K., 2004. Effect of respiratory homeostasis on plant growth in cultivars of wheat and rice. Plant, Cell & Environment 27, 853–862. doi:doi:10.1111/j.1365-3040.2004.01191.x.

Malagoli, P., Britto, D.T., Schulze, L.M., Kronzucker, H.J., 2008. Futile Na^+ cycling at the root plasma membrane in rice (*Oryza sativa* L.): kinetics, energetics, and relationship to salinity tolerance. Journal of Experimental Botany 59, 4109–4117. URL: http://dx.doi.org/10.1093/jxb/ern249, doi:10.1093/jxb/ern249.

Munns, R., Day, D.A., Fricke, W., Watt, M., Arsova, B., Barkla, B.J., Bose, J., Byrt, C.S., Chen, Z.H., Foster, K.J., Gilliham, M., Henderson, S.W., Jenkins, C.L., Kronzucker, H.J., Miklavcic, S.J., Plett, D., Roy, S.J., Shabala, S., Shelden, M.C., Soole, K.L., Taylor, N.L., Tester, M., Wege, S., Wegner, L.H., Tyerman, S.D., 2019a. Energy costs of salt tolerance in crop plants. New Phytologist 0. URL: https://nph.onlinelibrary.wiley.com/doi/abs/10.1111/nph.15864, doi:10.1111/nph.15864.

Munns, R., Passioura, J.B., Colmer, T.D., Byrt, C.S., 2019b. Osmotic adjustment and energy limitations to plant growth in saline soil. New Phytologist 0. URL: https://nph.onlinelibrary.wiley.com/doi/abs/10.1111/nph.15862, doi:10.1111/nph.15862.

Shabala, S., Chen, G., Chen, Z.H., Pottosin, I., 2019. The energy cost of the tonoplast futile sodium leak. New Phytologist 0. URL: https://nph.onlinelibrary.wiley.com/doi/abs/10.1111/nph.15758, doi:10.1111/nph.15758.

Teakle, N.L., Tyerman, S.D., 2010. Mechanisms of Cl^- transport contributing to salt tolerance. Plant, Cell & Environment 33, 566–589. URL: https://onlinelibrary.wiley.com/doi/abs/10.1111/j.1365-3040.2009.02060.x, doi:doi:10.1111/j.1365-3040.2009.02060.x.
